# An Update of the Appropriate Treatment Strategies in Anaplastic Thyroid Cancer: A Population-Based Study of 735 Patients

**DOI:** 10.1155/2019/8428547

**Published:** 2019-02-19

**Authors:** Nai-si Huang, Xiao Shi, Bo-wen Lei, Wen-jun Wei, Zhong-wu Lu, Peng-cheng Yu, Yu Wang, Qing-hai Ji, Yu-long Wang

**Affiliations:** ^1^Department of Head and Neck Surgery, Fudan University Shanghai Cancer Center, Shanghai 200032, China; ^2^Department of Oncology, Shanghai Medical College, Fudan University, Shanghai, China

## Abstract

**Background:**

Anaplastic thyroid cancer (ATC) responds poorly to conventional therapies and requires a multidisciplinary approach to manage. The aim of the current study is to explore whether aggressive treatment is beneficial, especially the appropriate extent of surgery in ATC.

**Methods:**

Patients diagnosed with ATC from 2004 to 2014 were identified from the Surveillance, Epidemiology, and End Results (SEER) database and included in our study.

**Results:**

A total of 735 ATC patients were identified. The two-year overall survival (OS) rates for stage IVA, IVB, and IVC patients were 36.5%, 15.6%, and 1.4%, respectively. By directly comparing eight treatment modalities, we found that surgery + radiotherapy (RT) ± chemotherapy was the most effective treatment strategy. surgery + chemotherapy and RT + chemotherapy had comparable results (hazard ratio (HR) = 1.461, 95% confidential interval (CI): 0.843-2.531, *P* = 0.177). Multivariate Cox regression analysis also showed increased mortality risk in patients with increased age (HR = 1.022, *P* < 0.001), tumor extension to adjacent structures (HR = 1.649, *P* = 0.013), and distant metastasis (HR = 2.041, *P* < 0.001), while surgery + RT (HR = 0.600, *P* = 0.004) and chemotherapy (HR = 0.692, *P* = 0.010) were independently associated with improved OS. Further analysis revealed that patients undergoing total/near-total thyroidectomy (TT) had superior OS to those receiving less than TT (*P* < 0.001). In subgroup analysis, the benefit of TT remained significant in patients with tumors larger than 4.0 cm (HR = 0.776, 95% CI: 0.469-0.887, *P* = 0.007), with adjacent structure extension (HR = 0.642, 95% CI: 0.472-0.877, *P* = 0.005), including trachea and major vessels, but not in patients with early phase local disease such as tumor ≤ 4.0 cm or tumor within the thyroid or with minimal extrathyroidal extension. Patients with very locally advanced disease or distant metastasis could not benefit from TT as well.

**Conclusions:**

In operable cases, surgery + RT ± chemotherapy was the optimal treatment modality. Otherwise, RT + chemotherapy was the appropriate strategy. However, TT was not beneficial for very early stage or metastatic ATC.

## 1. Introduction

Anaplastic thyroid cancer (ATC), although accounting for less than 0.2% of thyroid cancer, is one of the most lethal malignancies and accounts for up to 14-39% of total mortality in thyroid cancer [1–3] with a median survival of only five months [4, 5]. The clinical presentations of ATC are associated with a rapidly enlarging neck mass including dyspnea, dysphagia, neck pain, and hoarseness [6, 7]. About 15% of ATC patients present with extensive local invasion, and half of them had distant metastases at initial diagnosis [8].

ATC responds poorly to conventional therapies of thyroid cancer and requires a multidisciplinary approach under many circumstances. The combination of surgery, radiation therapy (RT), and chemotherapy is recommended to improve the locoregional and distant control of the disease [4]. Previous studies suggested that surgery with adjuvant RT improved survival, while other literatures recommended adding both RT and chemotherapy to the routine management of ATC [9–11]. However, due to the rare nature of this malignancy, most of these studies were single-institutional-based researches or results of clinical trials reported by high-volume institutions. Access to high-volume institutions is limited, especially for the elderly, ethnic, geographic, and racial minorities, and thus institutional studies may not be applicable to the general population [12].

Data from the population-based Surveillance, Epidemiology, and End Results (SEER) database, on the other hand, provides population-based data in the current status of ATC treatment. There were two previous studies based on the SEER database including 516 [13] and 261 [8] ATC patients, respectively, and summarized that the combined use of surgical resection and RT was independently associated with improved OS. They have provided the strongest evidence available in the multidisciplinary treatment of ATC so far. Unfortunately, the earlier version of the SEER database used by the authors above did not provide detailed information regarding the extent of surgery nor whether chemotherapy was delivered.

Based on the latest version of SEER database, we aimed to explore whether aggressive treatment (surgery, RT, and chemotherapy) is beneficial, especially the appropriate extent of surgery in ATC patients on a population-based level. Up to our knowledge, it is the first time to directly compare the results of eight treatment approaches and to reevaluate the role of total thyroidectomy in different clinical scenarios in ATC.

## 2. Materials and Methods

### 2.1. Patients

The SEER database of the US National Cancer Institute with anaplastic thyroid carcinoma (International Statistical Classification of Diseases for Oncology-3rd version code 8021) was used to identify ATC patients. We limited the cohort: (1) from 2004 to 2014 because of the lack of complete patient profiles in earlier cases, (2) patients with active follow-up during the study period until the cutoff follow-up date or the occurrence of endpoint event, and (3) patients with a known cause of death. Patients whose diagnoses were only based on autopsy reports or death certificate were excluded. The selection paradigm was presented in Supplementary Figure 1.

### 2.2. Definitions of Covariates

Patient baseline information (age, gender, and year of diagnosis), disease characteristics (primary tumor, lymph node metastasis, and distant metastasis), and treatment (surgery, RT, and chemotherapy) were included into analyses. The 7th edition of TNM staging system was used to stage the patients. Tumor size was recorded by “CS tumor size (2004+)”, and extension of primary tumor was divided into three categories according to “CS extension (2004+)” as (1) confined to thyroid, (2) minimal extension (thyroid capsule, strap muscles, and pericapsular soft tissue), and (3) adjuvant structures/organs (trachea, esophagus, recurrent laryngeal nerve, etc.). Surgery was defined as lobectomy with/without resection of the contralateral lobe while tracheotomy and excision biopsy were excluded. We further divided the extent of surgery by “RX Summ-Surg Prim Site (1988+)” into two parts: (1) less than total thyroidectomy (less than TT, including lobectomy ± isthmectomy ± partial removal of the contralateral lobe) and (2) total/near-total thyroidectomy (TT). Information of cervical lymph node management was extracted from the “RX Summ-Scope Reg LN Sur (2003+),” “regional nodes examined (1988+),” and “regional nodes positive (1988+).” The primary outcome of the present study was overall survival (OS), the duration of which was defined as the time from diagnosis to death from any cause.

### 2.3. Statistical Analysis

Cox regression models were used to identify risk factors contributed to OS and to compare the hazard ratios (HRs) and 95% confidential intervals (CIs) of different treatment modalities. Survival rates were estimated using the Kaplan-Meier method, and the significance of comparisons was calculated by the log-rank test. All statistical analyses were conducted with IBM SPSS version 24 (Chicago, IL, USA). A two-sided *P* value < 0.05 was considered to be statistically significant.

## 3. Results

### 3.1. Baseline Features of 735 ATC Patients

A total of 735 ATC patients diagnosed between 2004 and 2014 were identified from the SEER database and included into the analysis. The median age at diagnosis was 70 years old (range: 26-98). The majority of patients were female (460, 62.6%). The median tumor size was 6.4 cm. Of all patients, 362 had lymph node metastasis and 335 had distant metastasis (Table 1). The OS rates of the cohort were 10.7% and 8.1% at two years and five years, respectively. And the two-year OS rates for stage IVA, IVB, and IVC patients were 36.5%, 15.6%, and 1.4%, respectively (*P* < 0.001).

According to the treatment strategies, we categorize the patients into eight treatment groups: 159 patients did not receive surgery, RT, or chemotherapy (T1); 27 had only chemotherapy (T2); 188 had only surgery (T3); 19 had surgery + chemotherapy (T4); 80 had only RT (T5); 125 had RT + chemotherapy (T6); 56 had surgery + RT (T7); 141 had surgery + RT + chemotherapy (T8).

### 3.2. Multidisciplinary Approach Improved OS in ATC

Subsequently, we directly compared OS rates in eight treatment groups (Table 2). Chemotherapy alone (T2) or surgery alone (T3) or RT alone (T5) showed comparable outcomes (T3 vs. T2: HR = 1.122, 95% CI: 0.713-1.764, *P* = 0.691; T5 vs. T2: HR = 1.310, 95% CI: 0.822-2.090, *P* = 0.256; T5 vs. T3: HR = 1.163, 95% CI: 0.866-1.561, *P* = 0.316). Adding chemotherapy to RT or surgery was more effective than RT alone or surgery alone, while surgery + chemotherapy (T4) and RT + chemotherapy (T6) had comparable results (T6 vs. T4: HR = 1.461, 95% CI: 0.843-2.531, *P* = 0.177). surgery + RT (T7) revealed significantly decreased HRs compared with other treatments (T1-6); however, adding chemotherapy to surgery + RT did not seem to further improve the OS (T8 vs. T7: HR = 0.955, 95% CI: 0.670-1.361, *P* = 0.797).

**Figure 1:**
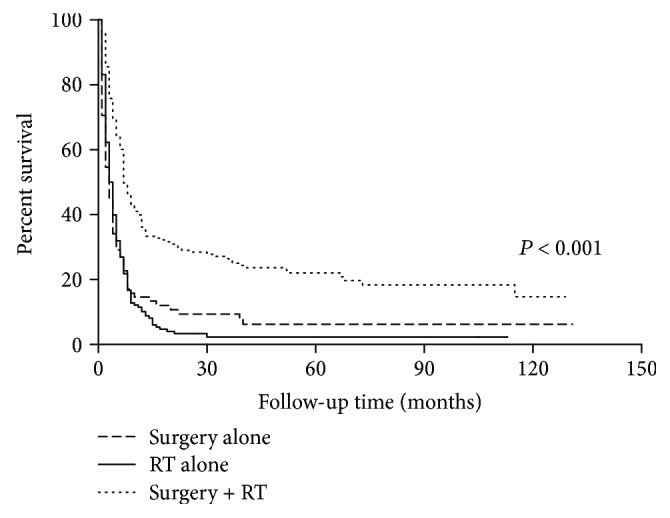
Surgery + RT could significantly improve overall survival in anaplastic thyroid cancer patients compared with surgery alone and RT alone.

Next, we limited the analysis into 316 patients who had locoregional treatments (surgery or RT). Patients in the surgery + RT group showed a significant survival benefit than other groups. The two-year OS in the surgery + RT group was 28.8% compared with 7.7% in the surgery-alone group (*P* < 0.001) and 3.0% in the RT-alone group (*P* < 0.001) (Figure 1). However, there was no statistical difference between the surgery-alone and RT-alone groups (*P* = 0.663). Using the multivariate Cox regression model (Table 3), we observed increased mortality in patients with increased age (HR = 1.022, 95% CI: 1.010-1.034, *P* < 0.001), tumor extension to adjacent structures (HR = 1.649, 95% CI: 1.113-2.445, *P* = 0.013), and distant metastasis (HR = 2.041, 95% CI: 1.532-2.720, *P* < 0.001). surgery + RT could significantly improve OS compared with surgery alone (HR = 0.600, 95% CI: 0.423-0.852, *P* = 0.004). Chemotherapy was also an independent prognostic factor for improved OS (HR = 0.692, 95% CI: 0.522-0.915, *P* = 0.010).

In 197 patients who had both surgery and RT, only seven had RT prior to surgery, 187 had RT after surgery, two had RT both before and after surgery, and one patient had both RT and surgery, but the sequence was unknown.

### 3.3. Surgery of the Primary Tumor

Since the detailed information regarding the regimens of chemotherapy and doses of RT was not accessible in the SEER database, we put emphasis on the exploration of the optimal surgical strategies in ATC in the setting of multidisciplinary treatment.

**Figure 2:**
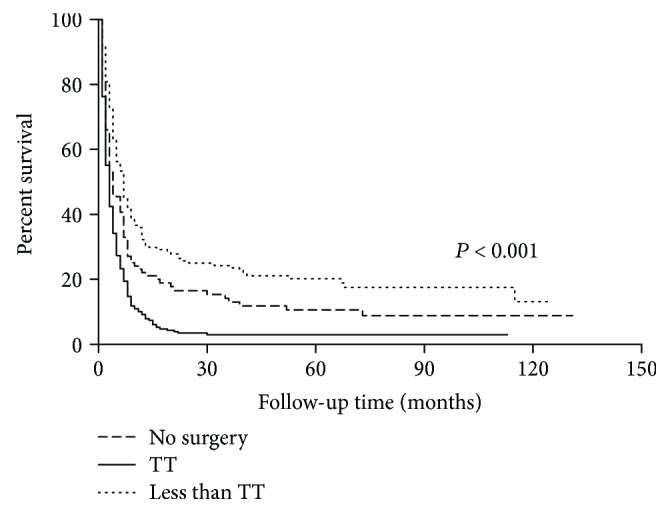
Surgery of the primary tumor site significantly improved the overall survival. Patients treated with total/near-total thyroidectomy (TT) showed superior survival compared with less than TT group and no-surgery group (*P* < 0.001).

In patients who received thyroid surgery, we further divided them into TT group (*N* = 195) and less than TT group (*N* = 130). These two groups were comparable in all baseline characteristics, such as gender, age, tumor extension, regional lymph node, and distant metastasis status. However, more patients had a tumor more than 6.4 cm in the less than TT group (40.0% vs. 27.7%, Supplementary Table 1). The two-year OS was 24.6%, 14.4%, and 2.4% in the TT group, less than TT group, and no-surgery group, respectively (*P* < 0.001, Figure 2).

**Figure 3:**
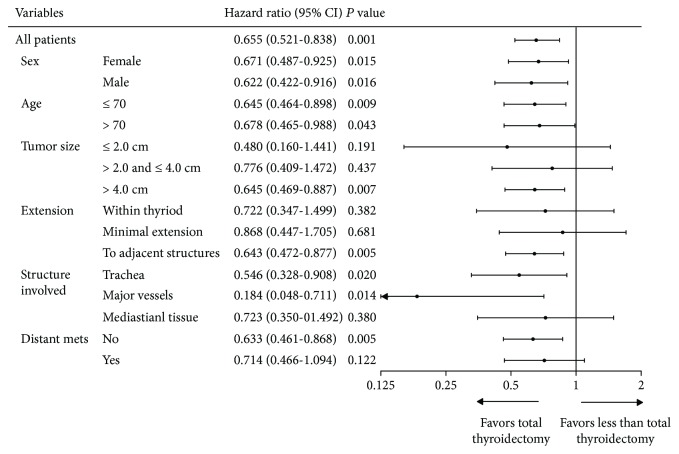
Subgroup analysis of total thyroidectomy versus less than total thyroidectomy using Cox regression models.

Cox regression analysis also showed that patients treated with TT had superior OS to patients treated with less than TT (HR = 0.655, 95% CI: 0.521-0.838, *P* = 0.001). The benefit of TT remained significant in all gender and age groups and patients with tumor size > 4.0 cm (HR = 0.776, 95% CI: 0.469-0.887, *P* = 0.007), with adjacent structure extension (HR = 0.642, 95% CI: 0.472-0.877, *P* = 0.005), including trachea and major vessels. Nevertheless, TT was not beneficial for patients with early phase local disease such as tumor ≤ 4.0 cm (*P* = 0.437), tumor within the thyroid (*P* = 0.382), or minimal extension (*P* = 0.681). Patients with very locally advanced disease (such as mediastinal tissue and prevertebral fascia extension, *P* = 0.380) or distant metastasis (*P* = 0.122) could not benefit from TT as well (Figure 3).

### 3.4. Surgery of the Regional Lymph Nodes

One hundred and seventy-one patients had cervical lymph node dissection, 32 had regional lymph node aspiration or biopsy, 517 patients did not have regional lymph node evaluation, and 15 were unknown. Among the 171 patients with lymph node dissection, 158 were recorded with the number of lymph node removed. The median of lymph nodes removed was 4 (range: 1-79). Pathology revealed that 54 patients had no lymph node metastasis, and the median of positive lymph nodes was 2 (range: 1-53). The median percentage of lymph node positivity was 23% (range: 0%-100%).

**Figure 4:**
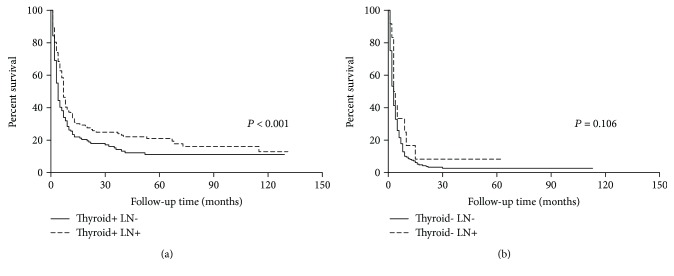
(a) In patients who had thyroid surgery, cervical lymph node dissection could improve patient survival, (b) while in patients without thyroid surgery, the survival benefit of lymph node dissection disappeared.

Compared with no lymph node dissection, patients with lymph node dissection had superior OS (HR = 0.503, 95% CI: 0.414-0.611, *P* < 0.001). Then we divided the patients into two groups according to whether they had thyroid surgery. Interestingly, although lymph node dissection could improve OS in patients who had thyroid surgery (*P* < 0.001, Figure 4(a)), for their counterparts without thyroid surgery, the survival benefit disappeared (*P* = 0.106, Figure 4(b)).

## 4. Discussion

Our study provided a perspective of the multidisciplinary treatment of ATC based on the SEER database. We reported that the OS rates of the cohort were 10.7% and 8.1% at two years and five years, respectively, which were comparable with previous population-based studies [8, 13]. In spite of emerging targeted therapies, the treatment outcome of ATC patients had made little progress over the decades and remained a lethal disease.

One of the most interesting results in the study was that we summarized the treatment of ATC and directly compare the results of eight treatment approaches. In patients who had treatment, the three most common strategies applied were surgery alone, surgery + RT + chemotherapy, and RT + chemotherapy. We found that in operable patients, surgery + RT ± chemotherapy was the optimal treatment, while in inoperable patients, RT + chemotherapy had superior results. These results implied that aggressive locoregional treatment (surgery + RT) should be applied to ATC patients whenever applicable. As previous studies indicated, extensive surgery with adjuvant RT was the optimal local regional treatment strategy in ATC, and long-term survival might be acquired in these cases, especially when the tumor was less invasive [14–16]. A recent study also suggested that initial intensive multimodal therapy was associated with improved ATC survival compared with palliative care [17]. Considering the patients' will and clinical status, if surgery + RT could not be delivered, surgery/RT + chemotherapy was the suitable option. Pezzi et al. showed that in unresectable ATC, RT dose was positively correlated with favorable OS, which highlighted inoperable ATC patients could still yet benefit from higher dose (60-75 Gy) of RT [18]. Since ATC is a rare and lethal disease, patients might die before planned treatments were delivered; only future prospective studies with comparable patient groups could verify our speculation based on the SEER database.

In cases with adequate locoregional therapy (surgery + RT), our analysis showed that adding chemotherapy could not further improve OS. Nevertheless, Rao et al. reported that up to 74% of patients had distant disease progression during treatment and there was a potential opportunity to improve outcomes with earlier initiation of systemic therapy [19]. One of the most promising results of chemoradiation to date was RT combined with doxorubibin ± taxanes or cisplatin [2]. Among these agents, doxorubicin was the most commonly used one, with a response rate of 22% [20]. Targeted therapies such as lenvatinib and dabrafenib plus trametinib (for BRAF (V600E) mutants) may provide a clinical benefit in ATC patients, and future studies are awaited to investigate whether new systemic treatment could benefit these patients [21].

Another highlight of the current study was that we reevaluated the role of TT and found that TT was not beneficial for all patients. For patients with locally restrained disease, the TT group showed nonsuperior OS than the control group. These results were in accordance with previous studies that complete resection (R0/R1) was associated with improved survival in ATC patients [22–24]. Since the data of R0/R1 resection was not provided by the SEER database, we could only deduce that TT was not necessary for accidentally identified or very early stage ATCs; meanwhile, complete resection of the tumor was enough for these patients. The rationale of TT, as indicated by the ATA guidelines, is based primarily on treatment recommendations related to the nonanaplastic component of the malignancy and was only deduced from the fact that approximately 20% of patients with ATC have coexisting DTC [4]. Given the complications of TT, lobectomy with negative margins seemed to be a more reasonable option. Of note, TT should be considered in clinically identified multifocal disease.

On the other hand, in patients with larger tumor size and adjacent structures extension, TT had a survival advantage due to the complete resection of the tumor which could not be achieved by less than TT. However, for patients with very advanced disease (such as distant metastasis), our data suggested that TT was not correlated with OS due to the extreme fatal nature of ATC. These were correlated with previous studies that there was no benefit of surgery in IVC patients [8].

Up to 49.3% of ATC patients presented with cervical lymph node metastases, still, the surgery of reginal lymph nodes was seldom discussed. Our findings suggested that cervical lymph node dissection could improve OS in patients with thyroid surgery. This result was consistent with multivariate Cox analysis of the current study and previous studies that lymph node metastasis was not an independent prognostic factor for OS in ATC patients.

The strengths of the current study were as follows: (1) unlike previous SEER-based studies, we were able to include more variables into our analyses owning to the update of the database since 2004; (2) we analyzed the scope of surgery of the thyroid and regional lymph nodes in ATC; and (3) for the first time, we directly compare all the treatment strategies of ATC. However, our research was limited by its retrospective nature, and the lack of more detailed information of RT and chemotherapy made it impossible to explore the optimal RT doses and chemotherapy regimens.

## 5. Conclusions

By analyzing 735 patients treated during 2004-2014, we identified that aggressive treatment could improve OS in ATC. In operable cases, surgery + RT ± chemotherapy was the optimal treatment option; otherwise, RT + chemotherapy was the appropriate strategy. However, the scope of thyroid surgery should depend on the scope of local disease. TT was not beneficial for very early stage or metastatic ATC compared with less than TT.

## Figures and Tables

**Table 1:** Baseline characteristics of 735 ATC patients.

Variables		No.	%
Age	Median 70 (IQR: 60-80)		
Gender	Female	460	62.6
	Male	275	37.4
Tumor size	≤2.0 cm	22	3.0
	2.1-4.0 cm	89	12.1
	4.1-6.0 cm	154	21.0
	>6.0 cm	297	40.4
	Unknown	173	23.5
Tumor extension	Confined within thyroid	89	12.1
	Minimal extension	73	9.9
	Extension to adjacent structures	439	59.7
	Unknown	134	18.2
LN involvement	N0	262	35.6
	N1a	71	9.7
	N1b	230	31.3
	N1 (NOS)	61	8.3
	Nx	111	15.1
Distant metastasis	No metastasis	344	46.8
	Distant LN only	11	1.5
	Distant metastasis ± distant LN	302	41.1
	Distant metastasis (NOS)	22	3.0
	Unknown	56	7.6
Year of diagnosis	2004-2007	214	29.1
	2008-2011	297	40.4
	2012-2014	224	30.5

ATC: anaplastic thyroid cancer; IQR: interquartile range; LN: lymph node; NOS: not specified.

**Table 2:** Direct comparisons between different treatment strategies in ATC patients using Cox regression models. There were eight treatment options: no treatment, chemotherapy alone, surgery ± chemotherapy, RT ± chemotherapy, and surgery + RT ± chemotherapy, and were numbered with T1, T2, T3,…, T8, respectively. Each column represented one treatment option. For instance, the contents in column one from the first cell to the last were the hazard ratio and 95% confidential interval of T2, T3,…, T8 compared with T1 as reference.

No treatment *n* = 159 (T1)	Chemo only *n* = 27 (T2)	Surgery only *n* = 118 (T3)	Surgery + chemo*n* = 19 (T4)	RT only *n* = 80 (T5)	RT + chemo*n* = 125 (T6)	Surgery + RT*n* = 56 (T7)
T2 vs. T1: 0.517 (0.331-0.807)						
T3 vs. T1: 0.551 (0.427-0.710)	T3 vs. T2: 1.122 (0.713-1.764)					
T4 vs. T1: 0.343 (0.199-0.594)	T4 vs. T2: 0.642 (0.333-1.237)	T4 vs. T3: 0.581 (0.336-1.003)				
T5 vs. T1: 0.639 (0.484-0.843)	T5 vs. T2: 1.310 (0.822-2.090)	T5 vs. T3: 1.163 (0.866-1.561)	T5 vs. T4: 2.139 (1.218-3.757)			
T6 vs. T1: 0.373 (0.289-0.483)	T6 vs. T2: 0.802 (0.511-1.260)	T6 vs. T3: 0.710 (0.541-0.930)	T6 vs. T4: 1.461 (0.843-2.531)	T6 vs. T5: 0.591 (0.441-0.792)		
T7 vs. T1: 0.239 (0.164-0.347)	T7 vs. T2: 0.447 (0.267-0.750)	T7 vs. T3: 0.418 (0.290-0.604)	T7 vs. T4: 0.686 (0.380-1.238)	T7 vs. T5: 0.319 (0.215-0.474)	T7 vs. T6: 0.463 (0.319-0.671)	
T8 vs. T1: 0.199 (0.150-0.263)	T8 vs. T2: 0.389 (0.246-0.614)	T8 vs. T3: 0.367 (0.277-0.486)	T8 vs. T4: 0.664 (0.386-1.142)	T8 vs. T5: 0.289 (0.212-0.393)	T8 vs. T6: 0.452 (0.342-0.597)	T8 vs. T7: 0.955 (0.670-1.361)
Reference: T1	Reference: T2	Reference: T3	Reference: T4	Reference: T5	Reference: T6	Reference: T7

ATC: anaplastic thyroid cancer; RT: radiotherapy; T: treatment; T8: surgery + RT + chemo*n* = 141.

**Table 3:** Multivariate Cox regression analysis of overall survival in ATC patients.

Variables	HR	95% CI	*P* value
Age	1.022	1.010-1.034	<0.001
Female	[Reference]		
Male	1.147	0.877-1.500	0.315
Tumor size	1.002	0.999-1.004	0.147
Confined within thyroid	[Reference]		
Minimal extension	1.372	0.839-2.244	0.208
Extension to adjacent structures	1.649	1.113-2.445	0.013
No LN metastasis	[Reference]		
N1a	0.933	0.616-1.413	0.743
N1b	1.220	0.912-1.632	0.181
No distant metastasis	[Reference]		
Distant metastasis	2.041	1.532-2.720	<0.001
Surgery	[Reference]		
RT	1.064	0.752-1.505	0.726
surgery + RT	0.600	0.423-0.852	0.004
No chemotherapy	[Reference]		
Chemotherapy	0.692	0.522-0.915	0.010

ATC: anaplastic thyroid cancer; RT: radiotherapy; HR: hazard ratio; CI: confidential interval.

## Data Availability

All the data used to support the findings of this study are included within the article and within the supplementary information files.

## Supplementary Materials

Supplementary Materials Supplementary Figure 1: data selection flow chart for anaplastic thyroid cancer patients from the SEER database. Supplementary Table 1: baseline characteristics of patients in the total/near-total thyroidectomy and less than total thyroidectomy groups.
