# Supplementary material for: An Update of the Appropriate Treatment Strategies in Anaplastic Thyroid Cancer: A Population-Based Study of 735 Patients
Source: Int J Endocrinol. 2019 Feb 19;2019:8428547. doi: 10.1155/2019/8428547 (PMC6399533; doi:10.1155/2019/8428547)

Supplementary Table 1. Baseline characteristics of patients in the Total/ near total thyroidectomy and less than total thyroidectomy groups.

	Less than total		Total/ near total thyroidectomy		*P*-value*
	N	%	N	%	
Gender					
Female	76	58.5	119	61.0	0.644
Male	54	41.5	76	39.0	
Age					
≤70	73	56.2	119	61.0	0.382
>70	57	43.8	76	39.0	
Tumor size					
≤6.4 cm	66	50.8	99	50.8	0.003
>6.4 cm	36	27.7	78	40.0	
Unknown	28	21.5	18	9.2	
Tumor extension					
Within thyroid	15	11.5	35	17.9	0.251
Minimal extension	19	14.6	32	16.4	
To adjacent structures	81	62.3	114	58.5	
Unknown	15	11.5	14	7.2	
Regional LN metastasis					
No	56	43.1	94	48.2	0.283
Yes	60	46.2	89	45.6	
Unknown	14	10.8	12	6.2	
Distant metastasis					
No	84	64.6	128	65.6	0.579
Yes	39	30.0	61	31.3	
Unknown	7	5.4	6	3.1	

LN: lymph node

* *P*-values were calculated by Chi-square tests.

Supplementary Figure 1.

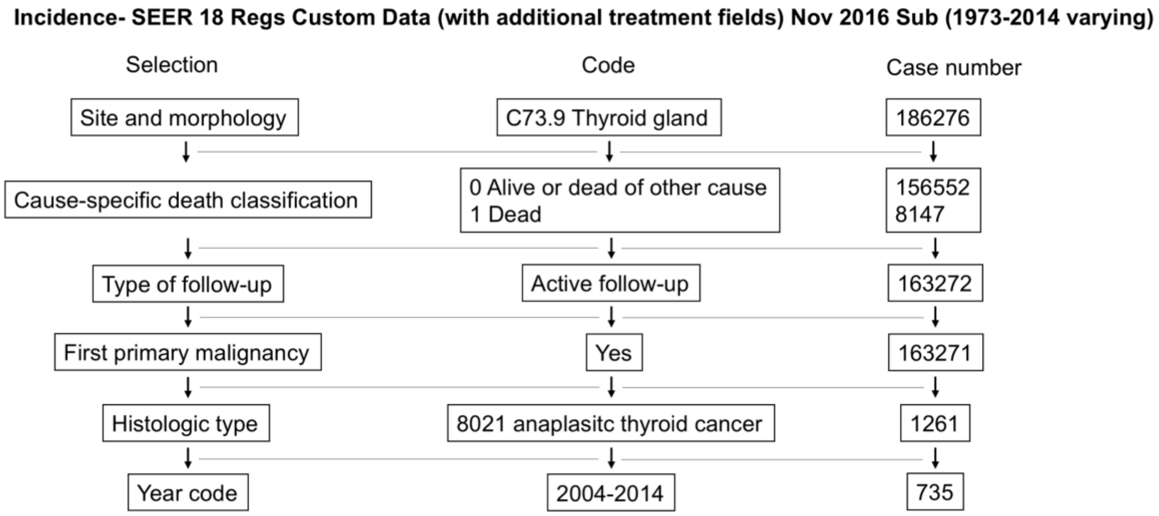

Supplement: Supplementary Materials — Supplementary Figure 1: data selection flow chart for anaplastic thyroid cancer patients from the SEER database. Supplementary Table 1: baseline characteristics of patients in the total/near-total thyroidectomy and less than total thyroidectomy groups. [file 8428547.f1.pdf]
